# A Study on Indigenous Knowledge on Animal Disease and Medicinal Plants Used in Animal Disease Management in Benatsemay Woreda, South Omo Zone, Southern Ethiopia

**DOI:** 10.1155/vmi/9951667

**Published:** 2025-04-12

**Authors:** Asrat Solomon Kenasew, Yebelayhun Mulugeta Tesfaye, Bakalo Basa Langana, Mulugeta Kusa Masha

**Affiliations:** Department of Veterinary Medicine, College of Agriculture and Natural Resource, Jinka University, Jinka, Ethiopia

**Keywords:** Benatsemay Woreda, disease, indigenous knowledge, medicinal plants

## Abstract

The study was conducted in South Omo Zone, Benatsemay Woreda of South Ethiopia Regional State of Ethiopia from July 2023 to June 2024 to assess and document the indigenous knowledge on animal disease and medicinal plants used to manage animal diseases. The study population was individual healers who were residents of Benatsemay Woreda with different sociodemographic characteristics. An ethnoveterinary botanical survey was conducted to gather information on the traditional usage of plants in the livestock healthcare system. Information was collected by direct interview with 40 known traditional healers and 8 key informants. Animals reared in the area include cattle, goats, sheep, poultry, equine, dogs, and cats. The animals were ranked based on their population number and importance to the livelihood of the family. Accordingly, cattle are ranked first based on both criteria. Diseases of animals prevailing in the study area were identified and ranked based on their morbidity and mortality rates as well. From the diseases of cattle, CBPP is ranked first based on both morbidity and mortality rates via pairwise ranking. Among the diseases of goats, CCPP and salmonellosis were important diseases. The prevalent sheep disease is ovine pasteurellosis with the highest mortality rate. Among poultry diseases, NCD, fowl cholera, and fowl pox were the dominant diseases in the area. Epizootic lymphangitis and rabies were the most important diseases in equine and dogs/cats, respectively. Traditional healers use different plant species to treat these diseases. Twenty-three plant species were collected, preserved, and botanically named. Root, leaves, seeds, and other plant parts were recorded that could be employed to treat sick animals. The most widely practiced administration of medicinal plant preparations was the oral administration of infusion.

## 1. Introduction

In many developing countries, livestock rearing/raising is one of the most important strategies to improve the living standards of the people [1]. Livestock production is important to the national economy in sub-Saharan Africa including Ethiopia, and it improves community livelihoods in rural areas [2]. Despite the abundance of cattle inEthiopia, the livestock subsector is generally less productive, its capacity is small, and its direct contribution to the national economy is minimal [3]. In addition to other factors, the poor health condition of its livestock has been one of the responsible factors for the low productivity [4]. It is believed that livestock-keeping communities possess a rich vocabulary to describe livestock health problems and local disease names with the corresponding clinical signs. Not only this, pastoral populations around the world hold complex and detailed ethnoveterinary knowledge, essential for the survival of their herds and securing their livelihood [5]. Ethnoveterinary medicine (EVM) is the use of medicinal plants (MPs), surgical techniques, and traditional management practices to prevent and treat spectrum of livestock diseases [6].

The livelihoods of the local communities heavily rely on livestock rearing, agriculture, and natural resources including MPs. However, environmental degradation, agricultural expansion, loss of forests and woodlands, overuse and overharvesting, fire, cultivation of marginal lands, overgrazing, and urbanization appear to be the major threats to the MPs of Ethiopia. Environmental degradation exacerbates the extinction of MPs and poses animal health risks in South Omo Zone. Deforestation, soil erosion, and water pollution not only harm the ecosystem but also compromise the health and well-being of the local communities. A growing population and the impacts of climate change put these areas under unprecedented pressure. Land degradation and extreme weather events such as droughts and floods lead to soil erosion, the loss of biodiversity, and decline of resilience to shocks. Land degradation manifests in these areas through the formation of dry valleys [7]. Furthermore, the animal health system in the South Omo Zone faces challenges in providing adequate service through a modern livestock health management system. Limited resources, infrastructure, and lack of trained personnel hinder the effective management of animal health issues, particularly those that require intensive treatment. Since traditional ways of treatment (EVM) appear to be a viable alternative approach to address the aforementioned issues in the study area, requirement for its preservation is undoubtful.

There are many MPs scattered all over the country, and a sizable number of them have been used for animal diseases. Rationalizing and validating the use of each MP requires thorough research [8]. Despite the significant role of MPs in treating both human and livestock ailments in Ethiopia, a very limited attempt has been made to explore, document, and promote these widely used MPs in the country [9]. It has enormous potential; however, this potential has not yet been exploited at the national level [10]. Furthermore, since cultural systems are dynamic [11], the skills will perish easily forgettable as most of the indigenous knowledge disseminated in Ethiopia is orally transmitted. Therefore, this knowledge has been disseminated from one generation to another without any documentation and without any preservation [12].

Although limited scientific research studies have focused on the use of indigenously known herbal remedies in animal healthcare in Ethiopia, many of the plants which are used in ethnoveterinary practice in these study areas were not exhaustively studied. Therefore, this study was designed to document the indigenous knowledge on diseases of animals and their MPs from this unexplored area.

## 2. Materials and Methods

### 2.1. Description of the Study Area

The study was conducted from July 2023 to June 2024 in Benatsemay Woreda, which is one of the woredas in South Omo Zone. The capital city Key Afer is located about 739 km from the capital city of Ethiopia, Addis Ababa, with a total land area of 3754 km^2^. Geographically, it is found at 5°01′-5°73′ N longitude and 36,°38′-37°07′ E latitude and at an altitudinal range of 1436–1553 m asl. The maximum and minimum monthly average temperatures of the area are 28.9°C and 17.3°C, respectively, with a total mean annual rainfall of 1167 mm. It is characterized by highly sloppy land features even more than 17% [13].

**Figure 1:**
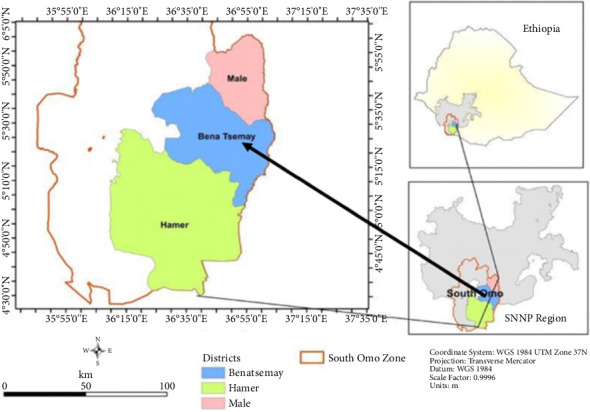
Map of Benatsemay Woreda [15].

The study area is characterized by semiarid and arid climatic conditions, with mean annual rainfall increasing from the extreme south lower part, with some 350 mm, to the upper part where it ranges to 838 mm. The rainfall is bimodal, with the long rains from April to June and the small rains in September and October [14]. The map of the study area is shown in Figure 1.

### 2.2. Study Design

A cross-sectional study was carried out from July 2023 to June 2024 to assess the indigenous knowledge on animal diseases and their MPs. Accordingly, traditional healers were selected purposively. The study population was individual healers who were residents of Benatsemay Woreda with different sociodemographic characteristics. This study included individuals of different sex, age categories, occupation, and marital status and those who were found on different educational levels. Besides this, the target population was interviewed with specific questions related to knowledge on animal diseases and MPs used to treat them.

### 2.3. Sample Size Determination

Key informants were selected based on the information gathered from the local people, while traditional healers were purposively selected. Ethnobotanical data were collected from these traditional healers using semistructured interviews and participant observations following [16]. The study population of the current study was comprised of purposively selected traditional healers with different educational levels, occupation, age, and sex groups. A preliminary study was performed before the actual research to identify the distribution of plants in different kebeles of the district. Unfortunately, the result showed that similar plants are distributed in different kebeles due to similar climatic conditions. But there are some kebeles with some differences in climate whichhave the plants found in other kebeles. Thus, the selected kebeles for this study were purposively selected as they possess almost all plant types in the district. Furthermore, livestock owners get medicine (MP) from these areas frequently. Thus, the required sample size for this study was estimated by considering the purposively estimated number of kebeles selected for this study and traditional healers in the respective kebeles of Benatsemay Woreda. For this study, 4 kebeles (namely, Kako, Chali, Luka, and Alduba) were selected. As mentioned above, as a result of the preliminary research, most livestock owners get MPs from these kebeles (mentioned by the key informants): 10 traditional healers and 2 key informants from each kebele; a total of 48 informants were purposively considered.

### 2.4. Method of Data Collection

#### 2.4.1. Ethnoveterinary Botanical Field Survey

**Figure 2:**
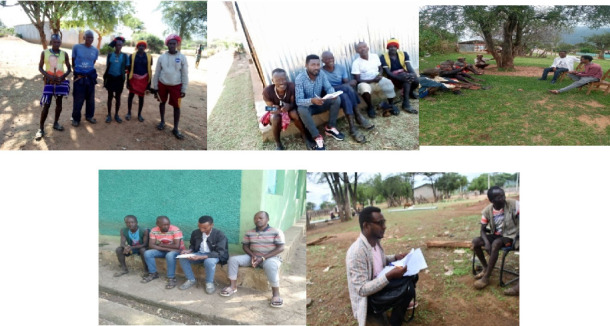
Ethnobotanical survey.

An ethnoveterinary botanical survey was conducted to gather information on the traditional usage of plants in the livestock healthcare system using a semistructured interview, observations, and field guided walks [16] with the traditional healers and key informants who were willing to share their indigenous knowledge. A prior communication was conducted with the local administrative and agriculture office for the objective of having affinity in the study area. A total of 48 individuals were purposively selected and interviewed for their knowledge on animal diseases and traditional medicine. In addition, the sociodemographic history of each respondent was recorded. An interview was presented to each purposively selected individual in a common local language (Amharic, Bena, and Tsemay). There was a brief discussion on the objective of the survey, and respondents were asked for their consent before the interview was commenced (Figure 2).

#### 2.4.2. Plant Collection and Identification

**Figure 3:**
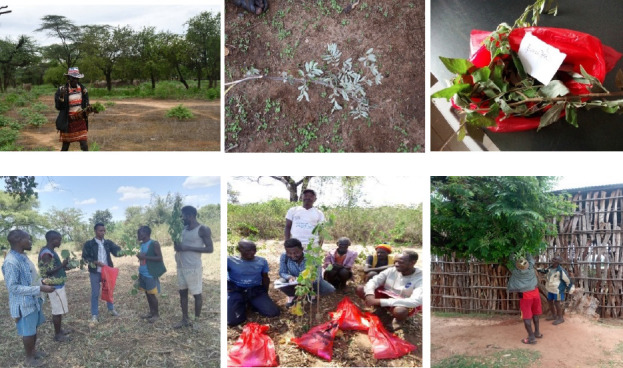
Collection of medicinal plants.

Specimens of plants identified by traditional healers and farmers for the treatment of livestock ailments were collected. Plant specimen collection was performed by including the vegetative part, leaves, and floral, fruiting, and/or seed parts as it was appropriate for taxonomic identification. During collection, information regarding habitat data, general information about the plant, and the geographical site of collection was recorded. The specimens were coded by their vernacular names and transported in plastic bags to avoid drying. After collection and drying, the specimens were identified botanically. Digital documentation of the specimen was considered for further storage as references (Figure 3).

#### 2.4.3. Participatory Epidemiology

**Figure 4:**
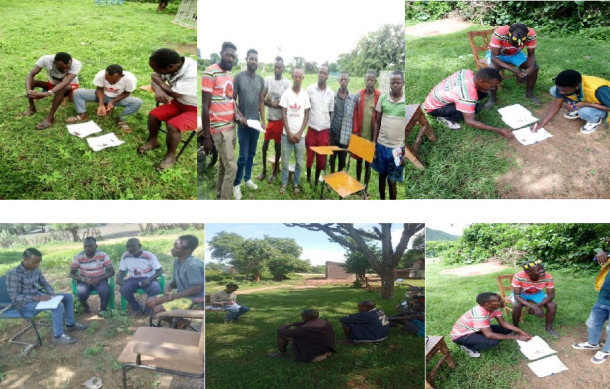
Participatory epidemiological survey.

Participatory epidemiology was held to identify the animals reared, rank the animals based on their population and their importance to household livelihood, and to identify their diseases and rank them based on morbidity and mortality in the study area. The respondents were 40 purposively selected animal owners who were said to have knowledge on animal diseases prevailing in the study area. The local or vernacular name of each disease was recorded in the languages of Tsemay, Bena, and some with Male and Ari languages. Proportional pilling and pairwise ranking were used to rank the diseases (Figure 4).

## 3. Data Management and Analysis

The ordinal data obtained from the participatory disease surveillance (PDS) were stored in Microsoft Excel 2013 and exported to SPSS Version 25 for analysis. Proportions (percentiles) and tables were used to summarize the collected ethnoveterinary knowledge on animal diseases and MPs data. A nonparametric statistical test, Kendall's coefficient of concordance (W), and Friedman's rank test were used. Kendall's coefficient was measure of agreement between informant groups on the data obtained from proportional pilling. Kendall's coefficient of concordance measures using the values between 0 (*no agreement*) and 1 (*complete agreement*). Agreement may be weak (*W* < 0.26), moderate (W between 0.26 and 0.38), and good (*W* > 0.38). Friedman's rank test was used to assess the agreements between informant groups on the data obtained from pairwise ranking. *p* value (< 0.05) was used to measure statistical significance of the variables considered.

## 4. Results

### 4.1. Animals Reared in the Study Area

**Figure 5:**
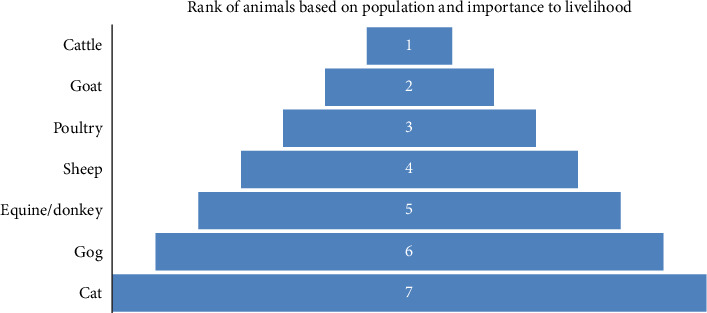
Rank of animals based on population and importance to household livelihood.

Animals reared in the study area include cattle, sheep, goats, equines, poultry, and small animals including dogs and cats. Based on the mean rank (Kendall's W test), cattle were ranked first in both population size and importance to household livelihood. Goats followed by poultry were the second and third important animals, respectively, in the area (Tables 1 and 2; Figure 5).

### 4.2. MPs Identified

**Figure 6:**
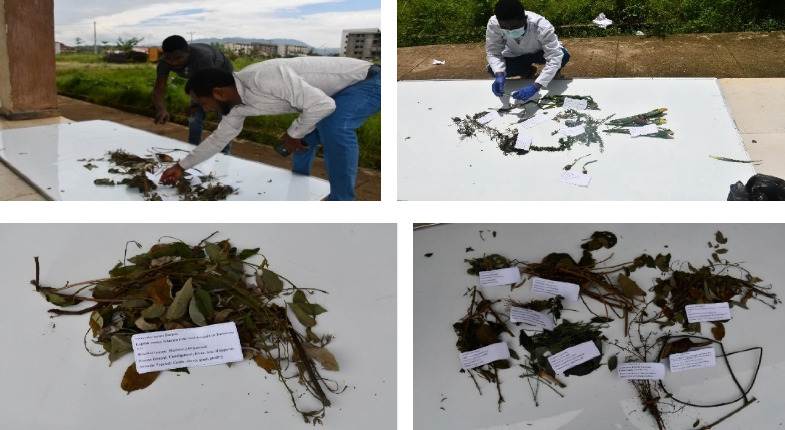
Naming of medicinal plants.

Considering the inadequate modern veterinary health services in the study area, indigenous community uses their own knowledge on the usage and management of animal diseases with the help of MPs. They have tremendous indigenous knowledge and skills in disease control and handling other specific conditions which they have developed over generations. A particular livestock owner was found to treat his or her own animals. In case the treatment trial of herdsmen failed to work, a healer in the area was consulted, either transported to the patients or the patient transported to the healer's homestead. In most cases, only ailments that the healers fail to treat were reported or brought to veterinary clinics. Twenty-three MPs were identified and given local (Vernacular), English, and scientific/botanical names (Appendix A). Among the plants collected from the study area, 48% were shrubs, 20% herbs, 20% trees, and the remaining 8% and 4% were vines and macrophytes, respectively. The dominant part of plants used for animal disease treatment was leaf. The way of preparation was mostly crushing/grinding. They apply them topically or administer them orally. The naming of the plants was performed via the help of AI technology (“picture this” application), authenticated by the botanist Yenesew Animaw of Jinka Agricultural Research Center, Jinka, Ethiopia (Figure 6). Furthermore, plants of world online (Royal Botanic Gardens, Kew) were referred to cross-check the accepted names. Voucher specimens have been stored at different herbaria such as Swedish Museum of Natural History Department of Botany (S), Centre de Recherche en Sciences Naturelles-CRSN-Lwiro Herbarium (LWI), University of Puerto Rico, Mayagüez Herbario (MAPR), Botanische Staatssammlung München (M), National Botanic Garden of Belgium (BR), Royal Botanic Garden Edinburgh (E), Barnes Arboretum at St. Joseph's University (ABFM), Institut National pour l'Étude et la Recherche Agronomique-Centre de Recherches Herbier (YBI), Muséum National d'Histoire Naturelle (P), Forestry Research Institute of Nigeria Taxonomy Section (FHI), United States National Herbarium, Smithsonian Institution (US), Linnean Society of London Herbarium (LINN), Royal Botanic Gardens, Kew (K), and Herbario Museo de La Plata (LP).

Among the MPs, *Mallotus philippensis (Lam.) Müll.Arg.* and *Eucalyptus globulus Labill* are abundant in 3 kebeles except Luka, whereas *Annona squamosa* L. and *Gymnanthemum amygdalinum (Delile) Sch. Bip.* are abundant in Kako and Alduba. According to the respondents, the most widely practiced administration of MP preparations was oral administration of the infusion. The list of MPs is presented in the Appendix A section.

### 4.3. Animal Diseases Identified in the Study Area

Diseases of animals reared in the study area were identified, and the first five diseases in cattle and three diseases in sheep and goats were ranked based on their morbidity and mortality rates in each kebele in the study vicinity. The mean rank of animal diseases in the study district based on morbidity and mortality was calculated with Kendall's W test (Tables 3, 4, and 5).

#### 4.3.1. Cattle Diseases Identified

Among diseases of cattle identified, infectious diseases like CBPP, blackleg, LSD, FMD, anthrax, trypanosomosis, and cowdriosis were identified in different kebeles. Based on the mean rank, CBPP was ranked first based on morbidity and mortality rates. Anthrax which is a zoonotic disease was ranked second based on the mortality rate in cattle.

CBPP, cowdriosis, LSD, blackleg, and trypanosomosis were ranked from first to fifth based on morbidity rate, respectively. CBPP, anthrax, cowdriosis, blackleg, and FMD were ranked the first five diseases on their mortality rate in the district. Test statistics for morbidity and mortality were employed, respectively (Tables 3 and 6).

According to test statistics of Kendall's *W* = 0.135, our informants in each kebele have weak agreement (*W* < 0.26) with each other. The value of 0.135 suggests a relatively low level of agreement among the 4 kebeles. The mean ranks tell us which disease was rated most favorably. The disease with the lowest mean rank (e.g., 3.13 is CBPP) was generally ranked as the most important or concerning disease by kebeles (Table 3).

The test statistics of mortality, Kendall's W test, indicates a low level of agreement (*W* = 0.124) among the 4 kebeles, and this low level of agreement is not statistically significant (*p*=0.812). The kebeles do not seem to have a consistent evaluation of the disease (Table 6).

The *p* value of 0.777 (*p* > 0.05) suggests that the observed level of agreement among the kebeles, as measured by Kendall's *W* = 0.135, and 0.124 are not statistically significant. We cannot conclude that the kebeles have a significant level of agreement in their rankings or evaluations of the diseases. Therefore, Kendall's W test indicates a low level of agreement (*W* = 0.135 and 0.124) for both morbidity and mortality, respectively, among the 4 kebeles, and this low level of agreement is not statistically significant (*p*=0.777 and 0.812) for both morbidity and mortality, respectively. The kebeles do not seem to have a consistent evaluation of the disease.

#### 4.3.2. Diseases of Goats

Similarly, among diseases of goats identified, infectious diseases such as CCPP, salmonellosis, Orf, foot rot, PPR, goat pox, cowdriosis, dermatophilosis, fasciolosis, and haemonchosis were identified in different kebeles. Based on the mean rank, CCPP was ranked first based on morbidity and mortality rates (Table 7).

#### 4.3.3. Diseases of Sheep

Diseases of sheep identified in different kebeles include ovine pasteurellosis, Orf, salmonellosis, cowdriosis, Coenurus cerebralis, brucellosis, fasciolosis, tick and mange mite infestations, and gastrointestinal parasites (Table 8).

#### 4.3.4. Diseases of Equine

As our respondents, the occurrence of diseases of equine is rare. Accordingly, trypanosomosis, epizootic lymphangitis, and verminous pneumonia are important diseases in the district (Table 9).

#### 4.3.5. Diseases of Poultry

**Figure 7:**
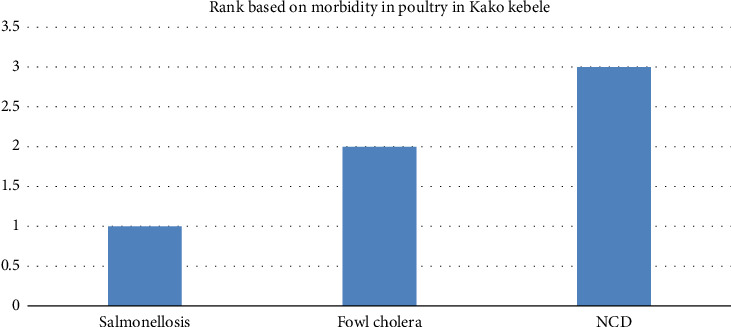
Rank of diseases in poultry by morbidity rate in Kako kebele.

Salmonellosis, fowl cholera, NCD, Marek's disease, fowl pox, and infectious coryza are important poultry diseases in the area, and there were no poultry in Luka. NCD disease which is a OIE listed disease is the deadliest disease in the area (Table 10; Figure 7).

#### 4.3.6. Diseases of Dogs and Cats

Rabies is the only disease of dogs and cats in the district especially the two kebeles with dog and cat population (Table 11).

### 4.4. Common Livestock Diseases and Their Traditional Medicaments

**Figure 8:**
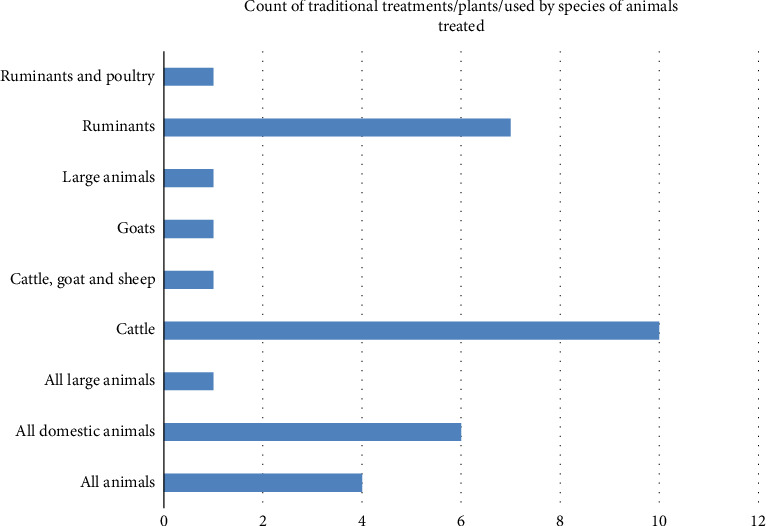
Count of traditional treatments/plants/used by species of animals treated (Appendix A).

There are many infectious and noninfectious diseases/conditions which are treated with MPs in the study area. These include anthrax, actinobacillosis, blackleg, bovine pasteurellosis, bloat, constipation, fever, retained placenta, wound, endoparasites, and ectoparasites. Some plants were used to treat two or more diseases/conditions, and others treat a single disease/condition. *Mallotus philippensis (Lam.) Müll. Arg.* was used to treat constipation, fever, and loss of appetite. Others like *Cissus quadrangularis* L. *and Malvastrum coromandelianum (L)* enhance immunity and promote growth and nursing calves after maternal death. The most frequently used parts of the plant include leaf (48%), followed by stem/stalk (24%), root (20%), leaf and stem (8%), and seeds to some extent. The most widely practiced route of administration of MP preparations was oral administration of infusion 16 (64%), followed by topical application of Paste 3 (12%), both topical and oral 2 (8%), intravaginal 2 (8%), and others including eye drops and hanging the plant at the gate door of animals. The formulations (a mixture of salt or sugar with the plant) were also adopted. Most (68%) of the livestock owners interviewed frequently use herbal preparations to treat their animals, whereas the remaining (32%) preferred the complementary use of both herbal preparations and conventional drugs. The number (count of) of traditional medicines and traditional treatments/plants used by a particular species of animal treated was investigated as well (Figure 8).

### 4.5. Specific Intervention Practices

The common specific practices of managing ailments and other conditions in the study area include physical and surgical interventions.

#### 4.5.1. Physical Therapy

The most widely used physical therapy was cauterization. It was commonly used for a number of diseases including lameness, foot and mouth disease, blackleg, hernia, abscess, chronic wound, and acute wound made by incision. A glown metal rod was used to perform cauterization.

#### 4.5.2. Surgical Interventions

##### 4.5.2.1. Dystocia.

The healers will thoroughly wash their hands and the perennial area of the animals with water. Then, they will perform manual traction or fetotomy depending on the defect identified.

##### 4.5.2.2. Vaginal and Uterine Prolapse

Treatment procedures required casting of animals and manual replacement of the uterus into the pelvis. The manipulative procedure adopted was suturing.

##### 4.5.2.3. Retained Fetal Membranes

It is a postpartum complication in most animals. The treatment procedures adopted by livestock owners include manual traction and use of big stone or wood hanged on the retained placenta.

##### 4.5.2.4. Castration

Closed castration was frequently carried out using a hammering material (blunt iron rod or wooden hammer) and a thick long stick. A strong sudden blow to spermatic cord with a hammering material will be given to crush.

##### 4.5.2.5. Dehorning and Hoof Trimming

These were carried out when there were hoof overgrowth and horn deformity with the aid of hot iron materials.

##### 4.5.2.6. Bone Setting

Several pieces of wooden materials were used. It was assembled over the fractured site with its center lying against the fracture and tied firmly and remains in place for weeks.

## 5. Discussion

This study revealed that the common domestic animals in the study area were cattle, followed by goats, sheep, poultry, equines, dogs, and cats. Cattle are ranked first based on the population and importance to household livelihood followed by goats. Cattle were ranked first by their importance to household livelihood in the study area as they are used for plowing, due to the economic value of cattle and their products (meat, milk, butter, manure, and skin), relative disease resistance, and social status. Similarly, goats provide meat and milk for the household; they tolerate harsh environment and high rate of production within short period of time and they appear to be used as emergency solutions. Since these animals are valuable for the community, disease prevention and control should be given priority to save their life, improve the production, and increase economic value as well.

EVM is the use of MPs, surgical techniques, and traditional management practices to prevent and treat spectrum of livestock diseases. The secret of information about EVM retained by traditional healers is relatively less susceptible to distortion but less accessible to the public [6]. The oral transmission of traditional knowledge on EVM gives rise to the possibility of incompleteness, omission, misrepresentation, or distortion of the original MPs as time goes on. Therefore, it presents an urgent need for being recorded and documented for future utilization [17]. Accordingly, 23 plants were recorded from the study area during the study period. But Bekele and Musa collected 18 plant species from the Chiro District. This difference might be attributed to the difference in the diversity of plants between the study areas. Among the plants, the leaf of *Solanum incanum* L. was used to treat bloat. This is in line with Sori et al. [18] who reported fruit of this plant was used to treat dermatophilosis in the Borana pastoralists, Ethiopia. Moreover, Mekonnen et al. reported in [19] that seeds of *Solanum incanum* L. were used for the treatment of leech infestations and colic in Dawuro Zone, Ethiopia. In Chiro, *Solanum incanum* L. was used for blood clotting and to treat skin diseases, blackleg, and colic [20]. According to the current finding, the leaf of *Gymnanthemum amygdalinum (Delile) Sch. Bip.* administered orally was used to treat trypanosomosis. This is similar to the report of Tekle [21] who reported the crushed seed of *Gymnanthemum amygdalinum (Delile) Sch. Bip* mixed with water and filtered for use of filtrate was used in the treatment of equine colic, pasteurellosis, and abdominal pain in Kochore District, Gedeo Zone, Ethiopia. Furthermore, Bekele and Mussa [20] reported that this plant is used for endoparasite treatment in Chiro. In the study area, *Achyranthes aspera* L. was used to treat endoparasites. But in Chiro it was used to treat blackleg [20]. This difference might be related to the difference in ethnoveterinary knowledge among the communities.

This study revealed that almost all parts of the plant (leaves, stems, roots, seeds, and flowers) were used in the preparation of traditional medicines. This is in agreement with the finding of Kaur and Mishra [22], who reported that bark, leaves, stems, flowers, roots, seeds, and fruits are used. Similarly, Bekele and Mussa [20] reported root, leaves, fruits, and other plant parts could be employed to treat sick animals.

Different routes of administration were employed in this study. Of these, oral, topical, intravaginal, and eye drops were the most commonly used routes of application. Oral route was the highest and most commonly used route. This is in agreement with the findings of Tekle [23] and Gebrezgabiher, Kalayou, and Sahle [24].

Some of the diseases mentioned by the respondents in this study indicated symptoms/clinical signs of disease. This is in line with the findings of Bekele and Mussa [20].

There was no agreement among kebeles during the ranking of animal diseases based on morbidity and mortality rates. Kendall's W test indicates a low level of agreement (*W* = 0.135 and 0.124), respectively. This might be due to differences in the ethnoveterinary knowledge among respondents, the difference in agroecology where animals graze, the prevalence of different diseases, morbidity rate, mortality rate, veterinary service and vaccination, and resistance among animals.

## 6. Conclusion and Recommendations

This study indicated that the high population of animal species is managed by the community in Benatsemay District. There were various infectious and noninfectious diseases of animals in the study area which require priority for their prevention. Since veterinary service and infrastructure are poor in the study area, indigenous knowledge on MPs has remained the most important livestock healthcare among rural farmers. There are plenty of MPs in the study area which play a great role in livestock disease treatment, mostly as a good alternative to conventional drugs which are expensive and even not accessible in the study area. But MPs were perceived to be easily available and effective on livestock disease treatment in the study area. Moreover, they have higher cultural acceptance than conventional veterinary medicine and are cheaper to get, and do not require special infrastructure for their administration. But some species of MPs are reported to have been threatened and decreasing by various human activities and climate changes in the study area. There were no conservation strategies for the MPs in the study area. Therefore, the availability status is questionable. If this trend continues, it will pose a risk to the poor farmers. As indigenous knowledge is fragile, not documented, and held by the traditional healers only in the study area, it will result in distortion and loss of this precious knowledge. Therefore, the government and other stakeholders should motivate people to conserve MP species and encourage farmers to adopt new technologies. Veterinary professionals should create awareness on animal disease prevention and establishment of veterinary service facilities at the nearest farmers in remote areas. As MPs are not only useful but also can be harmful due to lack of scientific proof of their efficacy and precise dosage which could produce toxicity, further study on the safety, dosage regimen effectiveness and efficacy on various infectious agents (bacteria, virus, fungi, protozoa, parasites, etc.), and the ingredients in the identified plants should be conducted by researchers.

## Figures and Tables

**Table 1:** Animals reared in the study area and their rank (proportional pilling).

Name of kebele	Animals kept	Local name	Rank based on population	Rank based on importance to household livelihood
Kako	Cattle	Waki	1	1
	Goat	Kole	2	2
	Sheep	Yati	4	4
	Equine/donkey	Ukili	5	5
	Poultry	Bocha	3	3
	Dog	Kasqi	6	6
	Cat	Wuri	7	7

Luka	Cattle	le'e	3	3
	Goat	Dhale	1	1
	Sheep	Eme	2	2
	Equine	Hare	5	5
	Poultry	Lukale	4	4
	Dog	Kare	6	6
	Cat	Wure	7	7

Alduba	Cattle	Waki	2	2

Alduba	Goat	Kole	1	1
	Sheep	Yati	3	3
	Equine/donkey	Ukili	4	4
	Poultry	Bocha	5	5
	Dog	Kasai	6	6
	Cat	Wuri	7	7

Chali	Cattle	Walasa/waki	1	1
	Goat	Kole/koli	2	2
	Sheep	Yati/mala/eti	3	3
	Equine/donkey	Ukili	5	4
	Poultry	Bacha/bacha'a	4	5
	Dog	Kaski/aski	6	6
	Cat	Wuri	7	7

**Table 2:** Mean rank of the animals based on population and their importance to household livelihood (from pair-wise ranking).

Animals kept	Rank based on population	Rank based on importance to household livelihood
Cattle	1	1
Goat	2	2
Poultry	3	3
Sheep	4	4
Equine/donkey	5	5
Dog	6	6
Cat	7	7

**Table 3:** Test statistics for morbidity.

Test statistics for morbidity	
*N*	4
Kendall's W^a^	0.135
Chi-square	3.245
Df	6
Asymp. sig.	0.777

^a^Kendall's coefficient of concordance.

**Table 4:** Mean rank of diseases of cattle based on morbidity and mortality (Kendall's W test, from pairwise ranking).

Name of disease	Mean rank (morbidity)	Rank	Mean rank (mortality)	Rank
CBPP	3.13	1	3.13	1
Cowdriosis	4.13	2	3.75	3
LSD	5.25	3	4.25	7
Blackleg	4.75	4	3.88	4
Trypanosomosis	3.50	5	5.50	6
Anthrax	3.25	6	3.50	2
FMD	4.00	7	4.00	5

**Table 5:** Cattle diseases identified in the study area and rank based on morbidity and mortality (proportional pilling).

Name of kebele	Diseases identified	Local name/vernacular name	Rank based on morbidity	Rank based on mortality
Kako	CBPP	Shompo	2	1
	Blackleg	Exa	3	3
	Antrax	Qunxa	4	2
	Trypanosomosis	Ficha	1	4
	LSD	Yeraza	5	5

Luka	CBPP	Shompo	2	1
	Blackleg	Exa	4	3
	Anthrax	Qunxa	3	2
	Trypanosomosis	Kusupho	1	4
	Cowdriosis		5	5

Alduba	Anthrax	Chula	3	1
	Trypanosomosis	Enako	4	2
	LSD	Burgudo	2	5
	FMD	Qundo	5	4
	Cowdriosis	Somba/uf etisa	1	3

Chali	LSD	Burdo	6	6
	Anthrax	Yeraza	3	1
	FMD	Toro	4	5
	Blackleg	Kunxa	2	2
	Trypanosomosis	Sirsira	5	4
	CBPP/bov.past.	Shompo	1	3

**Table 6:** Test statistics for mortality.

*N*	4
Kendall's W^a^	0.124
Chi-square	2.973
Df	6
Asymp. sig.	0.812

^a^Kendall's coefficient of concordance.

**Table 7:** Diseases of goats and their rank (proportional pilling).

Name of kebele	Diseases of goat	Local name/vernacular name	Rank based on morbidity	Rank based on mortality
Kako	Mange mite infestation/dermatophilosis	Kaxi	1	3
	CCPP	Shompo	2	1
	Salmonellosis	Arsa	3	2
	Orf	Bochi	5	4

Luka	Dermatophilosis/mange	Kaxi	1	3
	CCPP	Shompo	2	1
	Salmonellosis	Biratsa	3	2

Alduba	Cowdriosis	Somba/uf etisa	1	2
	PPR	Chadhasha	4	1

Alduba	Fasciolosis	Polo	2	4
	Haemonchosis	Koyo	3	3

Chali	CCPP	Piskile	1	1
	Goat pox	Tushta	2	2

**Table 8:** Diseases of sheep and their rank (proportional pilling).

Name of kebele	Diseases of sheep	Local name	Rank based on morbidity	Rank based on mortality
Kako	Ovine pasteurellosis	Shompo	1	1
	Foot rot	Tushita	2	4
	Coenurus cerebralis	Miti chishiya	4	2
	Orf	Bochi	3	3

Luka	Tick infestation	Shoka	1	2
	Orf	Bochu	3	3
	Salmonellosis	Harinti	2	1

Alduba	Cowdriosis	Somba/uf etisa	2	3
	Brucellosis	Koliho	3	4
	Fasciolosis	Polo	1	2
	PPR	Chadhasha	4	1

Chali	Ovine pasteurellosis	Piskile	3	1
	Mange mite	Kaxi	1	3
	GIT parasite	Kayo	2	2

**Table 9:** Diseases of equine and their rank (proportional pilling).

Name of kebele	Diseases of equine	Local name	Rank based on morbidity	Rank based on mortality
Kako	Trypanosomosis	Ficha	1	1

Alduba	Epizootic lymphangitis	Molisa	1	1
	Verminous pneumonia	Uf esa	2	2

Chali	Epizootic lymphangitis	Molisa	1	1

**Table 10:** Diseases of poultry and their rank (proportional pilling).

Name of kebele	Diseases of poultry	Local name	Rank based on morbidity	Rank based on mortality
Kako	Salmonellosis	Kachi/arsa	1	3
	Fowl cholera	Burdo	2	2
	NCD	Koche chirshidi	3	1

Alduba	Infectious coryza	Bohasa	1	1

Chali	Fowl pox	Tushta	1	3
	NCD	Koche wopsa	4	1
	Marek's disease	Roti kurdini	3	2
	Salmonellosis	Chawilipi edina	2	4

**Table 11:** Diseases of dogs and cats and their rank (proportional pilling).

Name of kebele	Diseases of dog and cat	Local name	Rank based on morbidity	Rank based on mortality
Kako	Rabies	Askiya baridine	1	1
Chali	Rabies	Hukuso/giskila/huksa	1	1

**Table 12:** List of medicinal plants used by traditional healers in the study area from July 2023 to June 2024.

Study kebele/place where plants were collected	Local name of medicinal plant	English name	Family name	Scientific name, author	Diseases treated	Plant parts used and route of administration	Method of remedy preparation and animals treated	Voucher number
Kako	Gedeq	Sugar apple	Annonaceae	*Annona squamosa* L., Linnnaeus [31]	Fascillosis, fever	Leaf^∗^	Infusion^c^	S08-820
	Gara	Bitter leaf	Asteraceae	*Gymnanthemum amygdalinum (delile) sch.bip.*, Linnnaeus [31]	Ectoparasites, bovine trypanosomosis	Leaf^∗^/^∗∗^	Chopping^a^	LWI119086782
	Chubisha	False mallow	Malvaceae	*Malvastrum coromandelianum (L). garcke*, Linnnaeus [31]	Enhance immunity and promote growth and nursing calves after maternal death	Leaf^∗^	Chopping^a^	MAPR13671
	Shafo	Balsa/corkwood	Malvaceae	*Ochroma pyramidale (cav.ex lam.) urb.*, Linnnaeus [31]	Constipation, retained placenta	Leaf/stalk^∗^	Infusion^b^	MAPR13813
	Dindo	Gooseneck loosestrife	Primulaceae	*Lysimachia clethroides duby*, Thunberg [26]	Chemical/plant/poisoning	Leaf^∗^	Chopping^a^	M0154059
	Boye	Cassava	Euphorbiaceae	*Manihot esculenta crantz*, Linnnaeus [31]	Bloat	Root^∗^	Chopping^a^	BR0000013321550
	Dhawako	Wild olive, silver berry	Elaeagnaceae	*Elaeagnus angustifolia* L., Linnnaeus [31]	Anthrax, blackleg, bovine [pateurollosis]	Leaf^∗^	Grinding^a^	E00408407

Luka	Qalqalko	Elephant bush	Didiereaceae	*Portulacaria afra jacq.*, Linnnaeus [31]	Bloat	Leaf^∗^	Grinding^a^	ABFM05249
	Kiriru	Veldt-grape, veld grape	Vitaceae	*Cissus quadrangularis* L., Linnnaeus [31]	Enhance immunity, promote growth and nursing calves after maternal death	Stem^∗∗∗^/^∗∗^	Grinding^a^	YBI109633885
	Najoo	Common passion fruit/purple passionfruit	Passifloraceae	*Passiflora edulis sims*, Linnnaeus [31]	Actinobacillosis	Stem, hanging/tying in the oral cavity	Used as it is^c^	P00455795
	Kariko	Sweet basil	Lamiaceae	*Ocimum basilicum* L., Linnnaeus [31]	Wound	Root^∗∗^	Grinding^c^	P00720609
	Argako/ret	True aloe, Chinese aloe	Asphodelaceae (liliaceae)	*Aloe vera (L.) burm.f.*, Holzworth [27]	Tick infestation	Leaf^∗∗^	Cutting the leaf and squeezing^c^	FHI0106026-0
	Kera	Cathedral cactus, abyssinia euphorbia	Euphorbiaceae	*Euphorbia trigona mill.*, Linnnaeus [31]	Prevention of all disease	Stem, hanging at entrance of animals	Hanging the stem at the entrance and pray^c^	US01432503
	Laqa	Common water hyacinth	Pontederiaceae	*Pontederia crassipes mart.*, Linnnaeus [31]	Wound	Seed/root^∗∗^	Grinding^c^	M0242220
	Chekenti	Umbrella thorn acacia	Fabaceae	Syntype of acacia spirocarpa hochst. ex A.rich., Linnnaeus [31]	Dermoid cyst	Leaf^∗∗∗∗^	Crushing^b^	MEL252177

Chali	Jalma	Ginger	Zingiberaceae	*Zingiber officinale* roscoe linnnaeus [31]	Dermoid cyst	Root^∗^	Crushing^b^	E00389845
	Choko (chali)	Wooly Dutch Man's pipe/wooly pipe vine/common Dutchman's pipe, wooly birthwort	Aristolochiaceae	*Aristolochia tomentosa sims.*, Linnnaeus [31]	Fever	Stem^∗^	Crushing^b^	LINN-HS1419-17
	Busanta	Prickly chaff flower	Amaranthaceae	*Achyranthes aspera* L., Linnnaeus [31]	Endoparasites	Root^∗^	Crushing^c^	YBI126645909

Chali	Dimba (chali)	Blue fountain bush	Lamiaceae	*Rotheca serrata (L.) steane$mabb.*, Linnnaeus [31]	Bloat	Leaf/stem^∗^	Crushing^a^	K000910222
	Garanti (chali)	Bitter apple	Solanaceae	*Solanum incanum* L., Linnnaeus [31]	Bloat	Leaf^∗^	Chopping^a^	K000414088
	Remit	Olive tree	Oleaceae	*Olea europaea* L., Linnnaeus [31]	Endoparasites	Root^∗^/^∗∗^	Crushing^c^	ABFM01852

Alduba	Bahirzaf	Tasmania blue gum	Myrtaceae	*Eucalyptus globulus labill.*, Labillardière [28]	Fever	Leaf^∗^	Crushing^c^	LP011034
	Baraza	Kamala tree/red kamala or kumkum tree	Euphorbiaceae	*Mallotus philippensis (lam.) müll.arg.*, Forster [29]	Constipation, fever, loss of appetite	Leaf^∗^	Crushing^e^	G00318137

*Note:* Source for voucher number: plants.jstor.org [30].

^∗^Oral drench.

^∗∗^Topical.

^∗∗∗^Intravaginal.

^∗∗∗∗^Eye drops.

^∗^/^∗∗^Oral/topical.

^a^Cattle.

^b^Ruminants.

^c^All domestic animals.

^d^Poultry.

^e^Ruminants and poultry.

## Data Availability

The data used to support the findings of this study are available at Jinka University, Jinka, Ethiopia.

## Appendix A. List of Medicinal Plants Used by Traditional Healers in the Study Area.

Note: As it is known that the plants have been known and utilized by indigenous peoples for centuries, they do not have a single discoverer or author in the way that scientific discovery might. The plants are used in various cultures before modern classification. However, the plants have been studied and documented by various botanists and researchers over time. Since Linneaus's classification laid a foundation for modern taxonomy, and while he did not discover the plant in the sense of being the first to encounter them, he is credited with naming and describing them scientifically. Therefore, at most the credit is given to Carl Linnaeus in this paper. His whole paper used as a title and described by Spencer [25] (Table A1).

## References

[1] Gebeyaw J., Kidanemariam F., Fesseha H. (2020). Assessment of Community Knowledge, Attitude and Practice towards Rabies in Mersa Town, Amhara Regional State, Ethiopia. *International Journal of Research Studies in Microbiology and Biotechnology*.

[2] Fesseha H., Abebe F. (2020). Assessment of Community Knowledge, Attitude, and Practice on Common Zoonotic Diseases in Jinka Town, Southern Ethiopia. *Journal of Gaz Medical Science*.

[3] Shitaye J. E., Tsegaye W., Pavlik I. (2007). Bovine Tuberculosis Infection in Animal and Human Populations in Ethiopia: A Review. *Veterinarni Medicina*.

[4] Giday M., Teklehaymanot T. (2013). Ethnobotanical Study of Plants Used in Management of Livestock Health Problems by Afar People of Ada’ar District, Afar Regional State, Ethiopia. *Journal of Ethnobiology and Ethnomedicine*.

[5] Volpato G., Lamin Saleh S. M., Di Nardo A. (2015). Ethnoveterinary of Sahrawi Pastoralists of Western Sahara: Camel Diseases and Remedies. *Journal of Ethnobiology and Ethnomedicine*.

[6] Oyda S. (2017). Review on Traditional Ethno-Veterinary Medicine and Medicinal Plants Used by Indigenous People in Ethiopia: Practice and Application System. *International Journal of Research-Granthaalayah*.

[7] GiZ (2023). Deutsche Gesellschaft für Internationale Zusammenarbeit (GIZ). [Online]. https://www.giz.de/en/worldwide/139466.html.

[8] Fekadu F. (2010). Ethiopian Medicinal Plants in Veterinary Healthcare A Mini-Review. *Ethioipian E-Journal for Research and Innovation Foresight*.

[9] Assen Y., Woldearegay M., Haile A. (2021). An Ethnobotanical Study of Medicinal Plants in Kelala District, South Wollo Zone of Amhara Region, Northeastern Ethiopia. *Evidence-Based Complementary and Alternative Medicine*.

[10] Berhanemeskel W., Teferra A., Ragunathan M. (2008). Ethnoveterinary Use of Medicinal Plants in Dabat District, Western Ethiopia. *Pharmacognosy Magazine*.

[11] Cunningham A. B. (2001). *Applied Ethnobotany: People, Wild Plant Use and Conser-Vation London and Sterling, VA*.

[12] Bullita S., Re G. A., Manunta M. D. I., Pilluza G. (2018). Traditional Knowledge About Plant, Animal and Mineral Based Remedies to Treat Cattle, Pigs, Horses and Other Domestic Animals in Amaediterrianian Islands of Sardinia. *Journal of Ethnobiology and Ethnomedicines*.

[13] Girma M., Getachew S., Tera A., Getaneh D., Birhanu T., SOFEDB (2014). (2016). Participatory on Farm Evaluation and Demonstration of 25% Crossbred (Boer X Woyito-Guji) Goats in Benatsemay District of South Omo Zone, SNNPR, Ethiopia. *Journal of Biology, Agriculture and Healthcare*.

[14] Benatsemay Woreda Agriculture Office (2023). *Annual Report*.

[15] Fesseha H., Eshetu E., Mathewos M., Tilante T. (2022). Study on Bovine Trypanosomiasis and Associated Risk Factors in Benatsemay District, Southern Ethiopia. *Environmental Health Insights*.

[16] Martin G. J. (1995). *Ethnobotany: A Conservation Manual*.

[17] Menzir A., Adeladlew T. (2020). A Review on Status of Ethnoveterinary Medicine and Challenges it Faces in Ethiopia. *International Journal of Veterinary Sciences and Animal Husbandry*.

[18] Sori T., Bekana M., Adugna G., Kelbessa E. (2004). Medicinal Plants in the Ethno Veterinary Practices of Borana Pastoralists, Southern Ethiopia. *International Journal of Applied Research and Veterinary Medicine*.

[19] Mekonnen M., Tessema F., Yilma M., Getachew T., Asrat M. (2016). Documentation of Ethno Veterinary Practices in Selected Sites of Wolaita and Dawuro Zones, Ethiopia. *Global Journal of Science Frontier Research*.

[20] Bekele A., Mussa A. (2009). Ethnoveterinary Practice in Chiro District Western Hararge, Ethiopia. *Pharmacologyonline*.

[21] Tekle Y. (2014). An Ethno-Veterinary Botanical Survey of Medicinal Plants in Kochore District of Gedeo Zone, Southern Nations Nationalities and Peoples Regional State (SNNPRs), Ethiopia. *Journal of Scientific and Innovative Research*.

[22] Kaur D., Jaiswal K., Mishra S. (2015). Ethnoveterinary Practices in India: A Review. *European Journal of Pharmaceutical and Medical Research*.

[23] Tekle Y. (2015). Study on Ethno Veterinary Practices in Amaro Special District Southern Ethiopia. *Medicinal & Aromatic Plants*.

[24] Gebrezgabiher G., Kalayou S., Sahle S. (2013). An Ethnoveterinary Survey of Medicinal Plants in Woredas of Tigray Region, Northern Ethiopia. *International Journal of Biodiversity and Conservation*.

[25] Spencer M. A. (2016). LINNAEUS, Carl. Species Plantarum 1753 Volumes 1 and 2, With an Introduction by W. T. Stearn and Appendix by J. L. Heller and W. T. Stearn, Plus Supplements by C. E. Jarvis. *Archives of Natural History*.

[26] Thunberg C. P. (1784). *Flora Japonica*.

[27] Holseworth R. E. (1996). *Aloe Vera: The Inside Story*.

[28] Labillardière J. (1800). *Novae Hollandiae Plantarum Specimen*.

[29] Forster G. (1786). *Florae Insularum Australium*.

[30] Jsor Global Plants (2025). https://Plants.jstor.org.

[31] Linnaeus C. (1753). *Species Plantrum. Vol. 1 and 2*.

